# Hepatitis B Vaccination and Screening Awareness in Primary Care Practitioners

**DOI:** 10.1155/2014/373212

**Published:** 2014-03-06

**Authors:** Adnan Said, Janice H. Jou

**Affiliations:** ^1^Division of Gastroenterology and Hepatology, University of Wisconsin School of Medicine and Public Health and William S. Middleton VAMC, Madison, WI 53705, USA; ^2^Division of Gastroenterology and Hepatology, Oregon Health Sciences University and Portland VAMC, Portland, OR 97239, USA

## Abstract

*Introduction.* The goals of *Healthy People US* 2020 have called for increased screening and vaccination of high-risk groups for Hepatitis B (HBV). *Methods.* We performed a survey of 400 randomly chosen primary care practitioners (PCPs) in Wisconsin to assess their knowledge, attitudes, and practices regarding screening and vaccination for HBV. *Results.* Screening rates of patients at risk of sexual transmission were low, with 61% of respondents stating that they screen patients who had more than 1 sex partner in 6 months and 86% screening patients with a history of sex with prostitutes. Screening rate for persons with a history of intravenous drug use was 94%. Children of immigrants were screened by 65%, persons on hemodialysis by 73%, and prison inmates by 69%. Screening increased with provider experience with HBV. Deficiencies in vaccination rates mirrored screening practices. Major barriers to screening were cost, someone else's responsibility, time constraints, or lack of knowledge. *Conclusions.* Without improved education and practices of PCPs about HBV screening and vaccination, the goals of healthy people 2020 regarding HBV will not be met. Barriers to screening and vaccination need to be addressed. Cost-effectiveness of alternative strategies such as universal vaccination under the age of 50 should be explored.

## 1. Introduction

Worldwide over 400 million people are carriers of hepatitis B; in the United States an estimated 1.25 million people are chronically infected and an estimated 51,000 new cases occurred in 2005 [1, 2]. Although the incidence of HBV has declined since the 1980s in all age groups, the decline has been slower in adults, particularly in males from ages 25–44 [2].

### 1.1. Vaccination Guidelines

Immunization Guidelines from the Centers for Disease Control (CDC) published in 1991 and updated in 1995 and 1999 call for universal vaccination of all persons younger than 18 years of age and adults older than 18 who are at risk for hepatitis B infection [3]. In December 2006, the Advisory Committee on Immunization Practices from the CDC released the first comprehensive statement on hepatitis B immunization since universal vaccination was advocated in 1991 [4]. These updated guidelines apart from reemphasizing the recommendations from previous iterations emphasized the importance of administering hepatitis B vaccination in primary care clinics as part of routine clinical care and to remove barriers for this care.

Although guidelines abound, 10% of practitioners are completely unaware of the existence of 78% of the guidelines [5] and current vaccination rates for adults and children at risk are less than optimal. A national survey of adults at risk for HBV found that only 30-31% had received the first dose of the HBV vaccine although 80% reported visiting a clinician during the past year [6]. There are missed opportunities for HBV vaccination in patients at risk as many of these subjects had multiple visits in one year. Data on vaccination rates for patients with specific risk factors tell a similar story.

### 1.2. Sexual Risk Factors

Among noninjection drug users, predictors of HBV seroconversion include females who engage in unprotected receptive anal sex, men who have sex with men (MSM), persons having a sex partner known for less than 6 months, and males and females who receive money or drugs for sex [7]. Vaccination rates in groups at risk of sexual transmission of HBV however remain low although recommendations for vaccination have been longstanding. In young men who have sex with men (MSM) the vaccination rates were only 9% with a prevalence of infection of 11% over the last two decades. Of this group, 96% had consistent contact with the health care system [8]. In recent years, the vaccination rates have improved somewhat although they are still far below acceptable levels. In a survey of MSM from 1999 to 2000, the vaccination rate for HBV was only 38.9% [9].

### 1.3. Immigration from Endemic Areas

The incidence of HBV is high in immigrants to the US from endemic areas of the world. Approximately 40,000 immigrants infected with chronic HBV are granted permanent residence in the United States annually [10]. A demographic group at particular risk is thus children of immigrants born in the United States, particularly those born before universal vaccination of neonates was instituted in 1991. Furthermore access to perinatal and neonatal healthcare is limited and these groups may not seek healthcare at these times reducing the opportunity for screening and vaccination and leading to increased rates of perinatal and horizontal transmission of HBV.

In a study of Korean-Americans from 1988 to 1990, males had a carrier rate of 8% for HBV while females had a rate of 4.4%. The rate of HBV carrier status in children of immigrants born in the United States was almost 3%. Furthermore, all mothers who had HBsAg and HBeAg had offspring who were HBsAg positive, underscoring the risk of perinatal transmission in immigrant populations [11]. In the Hmong population in Wisconsin who migrated from Laos, the overall prevalence of chronic HBV in Hmong children born in the United States between 1984 and 1989 was 14% [12]. Eleven percent of the HBsAg positive children were born to mothers who were HBsAg negative. Vaccination rates in this population vary widely from 12% to 86% [13, 14]. Household contacts of HBsAg carriers should be considered for screening and subsequent vaccination if they are susceptible [3, 4].

### 1.4. Incarceration

The risk in prison inmates is thought to be related to the high risk behaviors associated with this demographic including injection drug use and having multiple sex partners. In a correctional facility in Georgia, the prevalence of HBV infection was 2 percent, with an incidence in the facility in one year of almost 3 percent in inmates identified as high risk. The annual incidence of HBV in this facility (3579 per 100,000) was 120 times the national incidence. Additionally, clustering of unique HBV nucleotide sequencing in groups of newly infected inmates suggested that there was transmission between individuals at the correctional institution [15]. Investigation of two community outbreaks in Tennessee found that 60% of those infected had a history of incarceration. There was a prevalence of 4% for chronic HBV and 3% for acute HBV in this cohort.

### 1.5. Hemodialysis Patients

In dialysis patients the incidence and prevalence of HBV is high due to multiple parenteral exposures during dialysis and the immunosuppressed state associated with end stage renal disease [4, 16, 17]. Since 1982, HBV vaccination has been recommended for hemodialysis patients [18]. Although there have been significant improvements in the vaccination rates since that time, HBV vaccination rates in hemodialysis patients actually decreased in 14 of the 18 End Stage Renal Disease Network states, districts, and territories between 2001 and 2002. Furthermore, only 36.8–66.5% of hemodialysis patients in 2002 were vaccinated [19]. Dialysis centers are settings in which practitioners see high risk patients, and therefore further efforts should be undertaken to achieve 100% compliance with vaccination and to encourage periodic screening for HBV.

### 1.6. Role of Primary Care Practitioners

Primary care practitioners have a critical role in identifying and screening patients at high risk for acquisition for hepatitis B and subsequent vaccination of these patients [4]. However, our review of the medical literature suggests that little is known about primary care practitioners' awareness of guidelines for hepatitis B screening and vaccination, identifying the risk factors for transmission of hepatitis B, or experience of barriers to adherence to the guidelines [3, 20–22]. Our study sought to assess the knowledge, attitudes, and practices of primary care practitioners in Wisconsin regarding screening and vaccination guidelines for HBV and to identify barriers to these practices.

## 2. Methods

### 2.1. Study Sample

A master list of primary care physicians in Internal Medicine, Family Medicine, Pediatrics, and Obstetrics was obtained from the Wisconsin Department of Regulation and Licensing and used to select a random sample of 400 physicians from all over Wisconsin. A total of 166 practitioners responded to the survey. Forty-seven did not have a valid address or forwarding address, yielding a final response rate of 47%.

### 2.2. Survey Instrument

A 20-question survey (available upon request) was developed to assess clinician knowledge, attitudes, and practices in regard to hepatitis B vaccination and screening. Practitioners were asked about whether they offer HBV vaccinations, the number of patients seen with HBV, the number of patients vaccinated for HBV, screening practices, and barriers to screening. In addition, the practitioners were asked about their knowledge of the hepatitis B guidelines and attitudes toward HBV as a public health problem. Demographic data collected were practice type, patient population, years in practice, and country of residency training. The survey was tested for construct and content validity by expert review and by pretesting in a local group of PCPs.

### 2.3. Procedures

The study protocol was approved by the institutional review board of the University of Wisconsin School of Medicine and Public Health.

### 2.4. Data Collection

The first mailing was sent out without an incentive in November 2005. Included in the mailings were a self addressed stamped envelope, a survey with a code to permit tracking and follow-up, and a separate reply postcard. The post asked practitioners about their interest in participating in a future focus group to discuss barriers to hepatitis B vaccination and a CME program addressing hepatitis B vaccination and screening. A second mailing was sent in February 2007 to increase the response rate and included a five-dollar cash incentive.

### 2.5. Data Analysis

Question responses were initially reported as frequencies and percentages. For cross tabulations of discrete data chi-square statistics were used and a 2-sided *P* value of <0.05 was considered statistically significant. Binary logistic regression was used to examine independent predictors of discrete outcomes.

## 3. Results

### 3.1. Respondent Characteristics

Of the 166 respondents, 95 (57%) were in private practice, 25 (15%) were in Hospital-affiliated private practice, 13 (8%) were in a University Hospital Clinic, 25 (15%) responded “Other” (e.g., Community Health Clinic), and 8 (5%) did not answer the question. Thirty-one percent had been in practice for 0–10 years, 33% for 11–20 years, 25% for 21–30 years, and 11% for greater than 30 years. Forty percent of practitioners saw adults only, 40% saw both adults and children, and 20% saw children only.

More than half (53%) reported that they had not seen any patients with HBV infection in the past year, 40% had seen between 1 to 5 patients, 4% had seen 6–10 patients, and 3% had seen more than 10 patients with HBV. Residency training was completed in the United States for 97% of the respondents and internationally for three percent.

### 3.2. Current Practices for Screening for HBV Status

Overall, 59% felt that their current HBV screening practices were adequate. Practitioners were asked what proportion of their patients at risk for HBV were screened for infection in the past year: 19% responded that they screened all patients, 36% screened some but less than half, 31% screened more than half, and 14% did not screen any of their patients for HBV.

**Figure 1:**
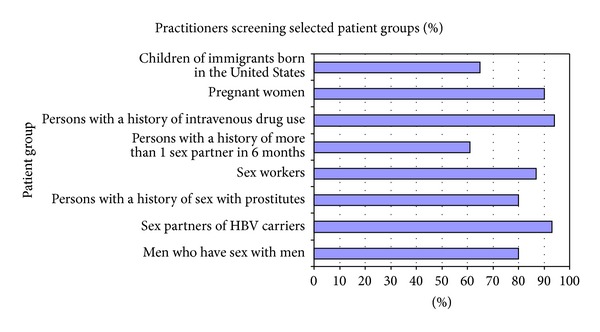
Percent of practitioners who would screen for HBV in selected groups.

**Figure 2:**
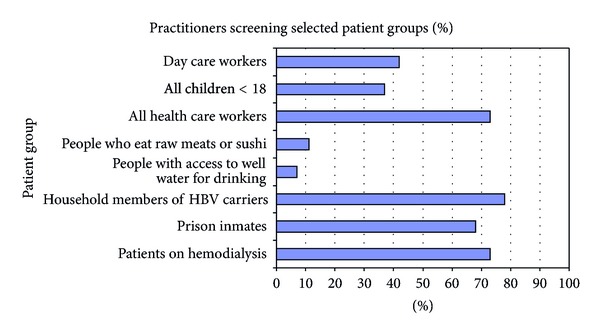
Percent of practitioners who would screen for HBV in selected groups.

The proportion of practitioners who screen various groups of patients is depicted in Figures 1 and 2. Ninety percent of practitioners stated they screen pregnant women. Sixty-one percent said that they screen persons with a history of more than one sex partner in six months and 80% screen men who have sex with men. The number of practitioners reporting that they screen persons with a history of sex with prostitutes was higher at 86% and for screening sex workers it was 87%.

Only 37% of PCPs stated that they screen everybody under the age of 18 and 65% screen US-born children of immigrants. Intravenous drug users are screened by 94% of the respondents. Prison inmates are screened by 69% and hemodialysis patients by 73%. Household members of HBV patients are screened by 78%.

Other groups not recommended in screening guidelines (distractor groups in the survey) such as day care workers are screened by 42% of providers, people drinking well water by 9%, and people eating sushi by 12%.

Screening of patients at risk for HBV increased with provider's experience with hepatitis B such that those who saw greater than 5 patients per year were more likely to screen at risk groups including sex workers, persons who have sex with prostitutes, sex partners of HBV carriers, prison inmates, and hemodialysis patients than were their counterparts in the study who saw less than 5 patients with HBV each year (*P* < 0.0001).

### 3.3. Vaccination Practices

Ninety-one percent of respondents were in a practice that offered HBV vaccination while 9% of the respondents were not. In the past year, 16% of practitioners had not vaccinated any patients for HBV, 14% had vaccinated 1–5 patients, 10% had vaccinated 6–10, and 60% had vaccinated more than 10 patients. When the practitioners were asked if they routinely asked their patients at risk for HBV if they had been vaccinated for HBV, 66% stated that they did, and 33% reported that they did not.

### 3.4. Knowledge of the HBV Guidelines and Etiology

**Figure 3:**
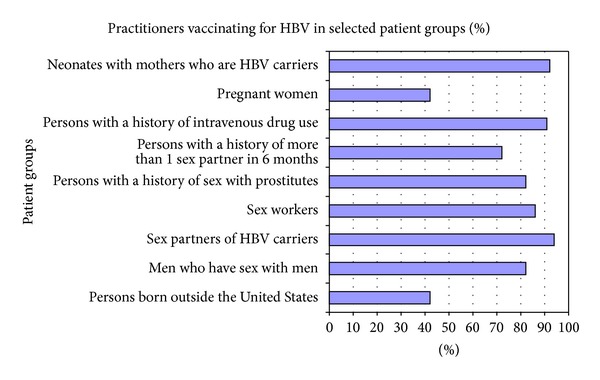
Percent of practitioners who would vaccinate for HBV in selected groups.

**Figure 4:**
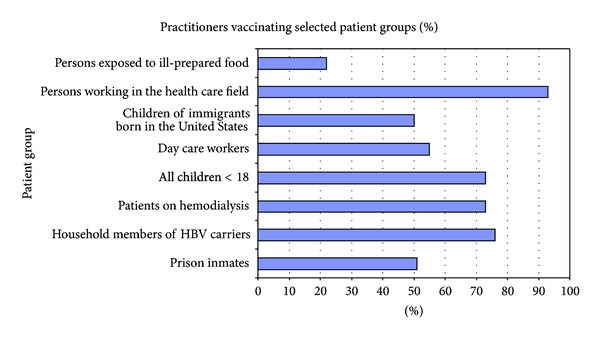
Percent of practitioners who would vaccinate for HBV in selected groups.

Of the groups identified by the CDC guidelines as populations who should receive vaccinations for HBV, several groups were correctly identified by a majority of respondents (Figures 3 and 4): persons with more than one sex partner in six months (72%), men who have sex with men (82%), intravenous drug users (92%), prison inmates (52%), and hemodialysis patients (72%). Persons who have sex with prostitutes would be vaccinated by 82% of providers and sex workers by 85%. Sexual partners of HBV positive patients would be vaccinated by 94%. Neonates born to mothers with HBV would be vaccinated by 92% of providers. A smaller number identified children 18 years old and younger (73%) as an appropriate group for HBV vaccination. Children of immigrants born in the United States would be vaccinated by only 50% of respondents.

Persons working in the health care field would be vaccinated by 92% of providers.

Other groups that are not recommended for vaccination such as persons exposed to ill-prepared food would be vaccinated by 22% of respondents, day care workers by 54%, and pregnant women by 42%.

The responses to the question asking for the most common cause of chronic viral hepatitis in the United States (HCV followed by HBV) were as follows: 4% hepatitis A (HAV), 39% HBV, 56% hepatitis C (HCV), and 1% hepatitis E (HEV). Another question was asked regarding the most common cause of chronic hepatitis worldwide and 16% replied that it was HAV, 70% HBV (correct answer), 13% HCV, and 1% HEV.

**Figure 5:**
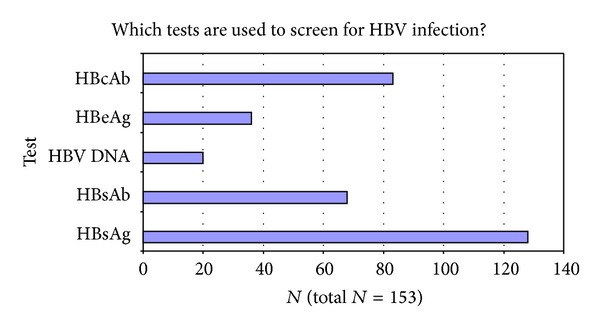
Which tests are used to screen for HBV infection?

Identification of serum tests for HBV screening are shown in Figure 5 with the majority of practitioners selecting HBsAg followed by HBcAb as the screening tests of choice.

### 3.5. Barriers to Screening

Regarding barriers to screening patients for HBV status, 11% percent responded that the cost of screening for HBV was too great, 7% felt that someone else was responsible for screening, and 3% felt that screening for HBV was not relevant to a patient's health care maintenance. Other barriers identified by respondents were lack of knowledge about HBV risk factors, vaccination and screening (9%), time constraints (5%), and forgetting or not making a point to ask (3%). Three percent felt that since they were pediatricians all of their patients must have been immunized and therefore it was unnecessary to screen their patients for HBV.

### 3.6. Attitudes toward HBV as a Public Health Issue

Practitioners were also asked how important HBV is as a public health problem in general with 48% responding that it was very important, 49% responding that it was somewhat important, and 3% felt that it was not important. In contrast, when asked whether or not they felt that HBV is an important public health problem in their own practice, only 18% believed it was very important, 44% somewhat important, and 38% not important.

## 4. Discussion

Despite being a vaccine preventable disease HBV remains prevalent worldwide. *Healthy People 2020 *called for increased vaccination rates for susceptible individuals [23]. Clearly, without adequate identification of patients who are at risk, HBV is unlikely to be eradicated [24]. Identification of all groups at risk for HBV is difficult as there are many modes of transmission including sexual, percutaneous, and mucosal exposures [10]. This requires an investment of time inquiring about these exposures and a process that would offer appropriate screening tests and vaccinations to eligible patients. Such a plan would rely heavily on the participation of primary care physicians who have the best opportunity for screening a broad cohort of patients.

Current screening practices emphasize the importance of risk factor identification particularly high risk sexual practices, intravenous drug use, and other risk groups such as prison inmates and hemodialysis patients. However, the cohort represented in this survey of PCPs was unable to consistently identify relevant risk factors. Intravenous drug use was identified as a risk factor by a majority of the respondents. The practitioners in our survey less consistently identified sexual risk factors for transmission of HBV; they were less attuned to HBV risks associated with being a child born in the United States to immigrants, a prison inmate, and a patient on hemodialysis.

Clearly these at risk populations need to be highlighted in educational screening and vaccination programs as well.

Even if risk factors are known, routine screening may not occur due to a variety of barriers. Many practitioners indicated that they were uncomfortable with inquiring about these risk factors from their patients, cost, time constraints, and lack of awareness and knowledge about groups at risk for HBV infection. In the medical literature, other barriers to adherence to practice guidelines are lack of self-efficacy, lack of outcome expectancy, inertia of previous practice, external barriers, and patient-related barriers. Many of those who responded to this survey did not feel that there were any areas of improvement in their practices in regard to hepatitis B vaccination and screening.

Funding is another barrier as many patients at risk for HBV are uninsured or underinsured.

### 4.1. Future Directions

There are options for interventions to increase HBV vaccination and screening rates. One would be educational programs for practitioners to increase knowledge of high risk groups for HBV to consider for screening and vaccination. Programs to implement the CDC guidelines can be established in medical institutions and primary care clinics. Additionally, correctional facilities are in great need of funding to vaccinate their inmates and dialysis centers need to vaccinate 100% of their susceptible patients. Follow-up surveys are being planned in the future to determine if over time HBV screening and vaccination rates have changed.

Since guidelines are difficult to keep up with and implement, an alternative strategy at a national level would be a reconsidering of the guidelines to vaccinate all persons under age of 50 to cast a wider net. The cost-effectiveness of this approach versus the current strategy (vaccinating everyone under 18 and adults at high risk) needs to be worked out.

The *Healthy People 2020* disease reduction goals have been established for achieving the prevention of HBV transmission in the United States. Disease reduction goals for infants and children include reducing the estimated number of chronic HBV infections in infants and young children to 400 cases and the number of new hepatitis B cases reported among persons 2–18 years of age to zero cases. Healthy People 2020 vaccination goals for infants and children include setting a target coverage level for infants of age of 0–3 days receiving the initial birth dose of hepatitis B vaccine to 85% and for children of age of 19–35 months completing the three-dose hepatitis B vaccination series to 90%.

Disease reduction goals for adults include reducing the rate of acute hepatitis B to 1.5 cases per 100,000 in persons of age of 19 years and older. Among adults in high-risk groups, disease reduction goals include reducing the number of cases of acute hepatitis B to 215 cases in injection-drug users and to 45 new infections among men who have sex with men [23]. Without educational intervention to increase awareness, and changing attitudes and practice, these goals will not be met.
